# Progress and ongoing conceptual challenges “on the way to integrative human neuroscience”–ten years after

**DOI:** 10.3389/fnint.2026.1792478

**Published:** 2026-05-15

**Authors:** Felix Tretter, Henriette Löffler-Stastka, Hans Albert Braun, Stephan Schleim, Brigitte Falkenburg, Heiner Hastedt, Georg Northoff, Thomas Fuchs, Boris Kotchoubey, Rainer Maderthaner, Andreas Draguhn

**Affiliations:** 1 Bertalanffy Center for the Study of Systems Science, Vienna, Austria; 2 Department of Psychoanalysis and Psychotherapy, Medical University of Vienna, Vienna, Austria; 3 AG Neurodynamics, Institute of Physiology and Pathophysiology, Philipps University of Marburg, Marburg, Germany; 4 Faculty of Behavioral and Social Sciences, University of Groningen, Groningen, Netherlands; 5 Department of Philosophy and Political Science, Technical University Dortmund, Dortmund, Germany; 6 Institute of Philosophy, University of Rostock, Rostock, Germany; 7 Mind, Brain Imaging and Neuroethics Unit, Institute of Mental Health Research, University of Ottawa, Ottawa, ON, Canada; 8 Karl Jaspers Chair, Phenomenological Psychopathology and Psychotherapy, Center for Psychosocial Medicine, University Clinics Heidelberg, Heidelberg, Germany; 9 Institute of Medical Psychology and Behavioral Neurobiology, University of Tübingen, Tübingen, Germany; 10 Institute for Psychology, University of Vienna, Vienna, Austria; 11 Institute for Physiology and Pathophysiology, Medical Faculty, University of Heidelberg, Heidelberg, Germany

**Keywords:** ecological perspective, free will, institutional limitations, neurophilosophy, systems psychology, systems science, theoretical neurobiology

## Abstract

Ten years ago, we published a position paper in this journal, criticizing reductionist claims of neurobiology related to mental disorders and important theoretical concepts like free will. Our interdisciplinary group of experts highlighted the need for and the challenges of integrating different approaches and system levels in neuroscience. We argued—and still argue—that such an integrative and multi-perspective approach is an important precondition for progress in the understanding and treatment of neuro-psychiatric disorders. We now review the progress towards an integrative neuroscience during the past decade in five steps: First, we examine the social and institutional context of brain research that has enabled tremendous technical developments and insights. Nevertheless, many research programs remain reductionist and fail to acknowledge differences between different system levels, their complex interactions, and domain-specific languages. We argue that scientific discourse largely lacks any critical account on the very nature of neurobiological explanations and interdisciplinary interfaces. Second, these conceptual weaknesses lead us to highlight the need for establishing an interdisciplinary neurophilosophy which tackles the challenging multiplicity of perspectives and approaches in modern neurosciences. The task is not just a collaboration between philosophers and neuroscientists, but rather the development of a critical philosophical stance within the neurosciences themselves. Third, based on this, we plead for the importance of the emerging science of complex systems, which is particularly helpful to integrate interdisciplinary knowledge and develop new strategies for modeling multi-level relations and phenomena. We suggest the application of systemic approaches in the mind sciences. Fourth, in line with this holistic view, we present an ecological perspective on human beings. The still dominating cephalocentric paradigm in neurosciences is severely limited without understanding the brain as a regulative organ in a situated organism and—in case of humans—an acting person “extended” to tools, technologies, and social structures. Fifth, in our final section, we illustrate our view using the debate about free will. We argue that any position respecting the complexity and irreducibility of mental phenomena will escape inappropriate reductionist and deterministic assumptions while fully acknowledging scientific evidence. We conclude with the demand for stronger efforts towards an institutionalized, interdisciplinary, systems-oriented neurophilosophy.

## 1 Some key characteristics of modern neuroscience

We published an interdisciplinary position paper addressing reductive claims of neurobiology ten years ago, with a focus on mental disorders and the question of “free will” (Kotchoubey et al., 2016). This paper in *Frontiers in Integrative Neuroscience* united experts from different fields including physics, neurobiology, psychiatry, psychology and philosophy. Our aim was to raise awareness within the neuroscientific community for the necessity and challenges of a methodological, conceptual and theoretical integration in neuroscience which, we believe, is an important precondition for transformative progress in the understanding and treatment of neuro-psychiatric disorders. We suggested a more active discussion of conceptual questions within and between disciplines and demanded more active efforts towards common theoretical foundations in psychology, cognitive and systemic neurosciences, psychopathology and psychiatry (i.e., sciences of the mind).

The 10th anniversary of our publication seems to be a good occasion to revisit this issue. We have witnessed tremendous technical developments, impressive new insights into mechanisms underlying cognition and behavior, and a massive accumulation of data at all system levels. However, several basic epistemic and pragmatic problems seem to persist.

Here we review recent developments and persisting challenges. We start by elucidating several key characteristics of modern neurosciences: the social and institutional context of brain research; the trend towards big data, and the conceptual state of theoretical neuroscience (section 1). We then explain and highlight the need for philosophical reflection, including a meta-scientific perspective (section 2). From there, we emphasize the importance of systemic thinking as an integrative approach (section 3). This leads towards the demand for systems psycho(patho)logy (section 4) and the need for a complementation of classical sciences by a phenomenological and ecological perspective (section 5). Finally, we exemplify the importance of an integrative approach by examining the debate on free will (section 6). We conclude the paper by demanding stronger efforts towards an institutionalized and interdisciplinary neurophilosophy, rooted in the *philosophy of mind* as well as the *philosophy of (interdisciplinary) science*.

### 1.1 Institutional aspects–upscaling science

Modern science, including neuroscience, is increasingly driven by technological progress and large-scale projects, with increasing influence of financial resource allocation. Progress is impressive, not at least due to modern recording techniques and manipulative approaches. Large-scale research projects (“big science”) have entered neuroscience, adding unprecedented, powerful data sets. On the other hand, the development can create a polarity between small-scale, hypothesis-driven and person-centered science on one side, and large research consortia with their peculiar mechanisms of fundraising and public communication on the other (Knorr-Cetina, 1999). The latter initiate massive research programs with typical features of “big science” (Dorsey et al., 2006; Editorial, 2014): major, broadly understood and accepted aims, promises for applications, multiple participating institutions, large budgets, strong standardization of data, industrial-style scientific work, large numbers of contributing individuals, and a focus on overarching aims rather than individual contributions.

The trend toward big science in the life sciences bears chances and opportunities, but also several challenges and potential pitfalls. Large-scale research depends strongly on resource allocation by major industrial or political institutions. These decision processes increase the weight of non-scientific arguments in determining research aims and strategies, on the costs of a merely science-guided process. Political agenda setting can stimulate worldwide research programs with unprecedented size and efficacy. However, this positive development may come at the costs of a thorough reflection about research aims and limitations, at least before facts are set. Prominent initiatives like the “decade of the brain” in the 1990s and the “human brain project” in the 2000s raise high expectations which might not be met by the results, in particular with respect to clinical applications (see below). A novel and exciting development is the emergence of large-scale digital infrastructures and data collections (e.g., Allen Institute, 2026). Such well-curated corpora constitute entirely new resources of unprecedented completeness and depth. With their precisely defined quality standards and public accessibility they bear great chances for discovery of new correlations, efficient data use and acceleration of research on a worldwide scale. Such “big science” projects may, however, reduce attention for the ongoing parallel and diverse work of scientists worldwide. However, pluralism of methods, model systems and hypotheses is particularly important in biomedical research given the large diversity of organisms, research traditions and phenomena. The dominance of funding and public awareness for large-scale projects is particularly problematic in a field which lacks a clear conceptual framing, like the neurosciences (see below and Frégnac, 2017). Within scientific institutions, the rise of large-scale projects fosters the development of new, industry-like working conditions, some of which might bear great chances.

A problematic aspect is the central role for media and politics in enabling and stabilizing the current trend toward “big science” in biology and medicine. Society seems willing to “follow this kind of science,” even if Robert Merton’s principle of “organized skepticism” is violated (Merton, 1942). The importance of unorthodox thoughts and approaches for scientific progress has been pointed out by Kuhn (1962), building upon earlier work emphasizing the conceptual differences between “old” and “new” scientific frameworks and, hence, the unpredictable nature of scientific progress (Fleck, 1935; see also Feyerabend, 1975). Approaches like “Critical Neuroscience” (Choudbury and Slaby, 2011) and “Sociology of Neuroscience” (Pickersgill and van Keulen, 2011; Pickersgill, 2023) offer important perspectives on the contextual conditions of scientific discourses and discoveries. They may help developing strategies for maintaining a good balance between powerful large-scale approaches and diverse small-scale projects. They cannot, however, replace additional reflections within a genuine philosophy of science (see section “2 Philosophy of neuroscience–key issues”).

### 1.2 The ongoing challenge of translation–Are more data more knowledge?

Insights into the human brain and its disorders have been a main driving force for medical progress since the rise of modern neurosciences toward the end of the 19th century. This development has been strongly boosted by modern neurosciences, opening the chance for precise, individualized diagnostics and therapy of neuro-psychiatric disorders. Particularly promising developments comprise genetic screening and stratification strategies (Grotzinger et al., 2026), pharmacogenomics (Pardiñas et al., 2021), massively parallel neuronal recordings (Urai et al., 2022; Lee et al., 2024), opto- or chemogenetic manipulations (Meron Asher and Goshen, 2025). A large portion of research in humans follows a correlational approach between mental and neuronal phenomena. Causal analysis of neuronal mechanisms underlying mental processes have been strongly boosted by opto- and chemogenetics but are largely confined to animal experiments. A remarkable exception are targeted neurostimulation techniques (Soleimani et al., 2025) which are increasingly used in psychiatric, rather than exclusively neurological diseases. The full therapeutic potential of these techniques remains to be elucidated, especially regarding neurological or psychological side-effects as well as the long-term stability of electrical stimulation approaches (Brandt et al., 2015). Another remarkable development comes from procedures which construct a closed circle between mental processes and neuronal activity: biofeedback (“neurofeedback”) and brain-computer interfaces (BCI). In neurofeedback, participants learn to control some neurophysiological parameter (e.g., an EEG amplitude, or the activity of a particular brain structure), which results in changes in their behavior and health. Human-learning BCI uses the same principle (Birbaumer et al., 1999) while in machine-learning BCI a computer learns to recognize useful patterns of brain activity and translates them into adaptive movements. In all such designs, physiological variables are driving functions for behavioral (psychological) effects. With this, they complement traditional experimental designs in human cognitive neuroscience and may help revealing causal relationships between brain activity and mental functions. Unfortunately, results from neurofeedback and BCI studies have not yet exerted a major impact on theoretical concepts of brain and mind - an urgently needed further step of integration. As a note of caution, it remains to be explained how the processes described in mental terminology (“needs,” “desires,” “urges,” “wishes,” “intentions” etc.) are related to the processed described in neural, hence physical and biological, terms (see chapters 4–6).

Besides all scientific breakthroughs, only few of the new approaches have been translated into improved diagnostics or treatment of large numbers of patients. In clinical psychiatry, not only the brain-based understanding of mental illnesses but also the diagnostic and therapeutic practice have not made essential progress during past decades, notwithstanding numerous incremental advances. Diagnostic schemes are mostly symptom-based and categorical, rather than referring to neurobiologically defined disease entities (American Psychiatric Association, 2013). The two most prominent innovations in psychopharmacology, ketamine in depression and psychedelics in various conditions, came about by empiric approaches, rather than by theory-driven and conceptually guided research. Impressive practical results may, however, come increasingly from artificial intelligence (AI) and machine learning which promise to support continuous, low-threshold assessment of individual patient’s state, refined data-based diagnosis and stratification, and individualized therapy (Opel and Breakspear, 2026). Again, the enormous potential of these techniques hast to be weighed against potential undesired effects like false positive diagnoses, insufficient control of therapeutic processes by medical personnel etc. A particularly important question refers to the very nature of artificial intelligence. Is it based on limited and semantically blind operations on large sets of data, or does it reason or/and produce explainable results in a sense akin to human cognition? Will explainable AI tools help us understanding fundamental mechanisms of normal or pathological brain functions? Might technical artifacts reach consciousness and/or genuine agency? The debate around these questions is complex and did not yet reach consensus (Seth, 2025; Butlin et al., 2023; Chowa et al., 2026). From a more practical point of view it is feasible that modern neurotechniques and robotics may constitute a leap in neuro-psychiatric therapy, akin to modern assistance devices for blind and paralyzed persons (Savage, 2019). These may, in turn, inspire new insights into the function of the underlying neuronal systems themselves.

In conclusion, we acknowledge that proper usage and analysis of big data sets may increasingly drive innovation. It should be noted, however, that evidence generated in this way is primarily correlational and does not provide explanatory knowledge and mechanistic understanding. Nevertheless, causal assumptions are often made based on coincidence measures such as correlation analysis, adjacency matrix analysis and procedures of AI which help extracting essential features which were hitherto hidden in complex data sets. These techniques lead to a more precise description and categorization of phenomena and may yield better stratifications of patients. They do not, however, enable a solid explanation and understanding (Leonelli, 2019). For these reasons, results from (big) data-driven approaches need conceptual interpretations, which implicate theoretical considerations. To put it in another way: big data should be integrated into theoretical frameworks. Therefore, a renewed programmatic call for the promotion of theory-oriented neuroscience seems justified.

### 1.3 Theoretical neuroscience - from areas to predictive networks, but what then?

Since the 2000s, the field of theoretical neuroscience has ultimately led to formal, neurobiologically grounded theories of consciousness, arguably one of the big enigmas in our field, and often regarded as inaccessible to neuroscientific explanations (Chalmers, 1995). Examples for such approaches are: Global Neuronal Workspace Theory (GNWT; Dehaene and Changeux, 2011), stating that consciousness arises from brain-wide extension of a dominant local network process; Recurrent Processing Theory (RPT; Lamme, 2010), proposing that consciousness arises from feedback of information to sensory processing systems; Predictive Processing Theory (PPT) combined with the Free Energy Principle (FEP) emphasizing the interplay between top-down processing of neuronal predictions (priors) and bottom-up processing of sensory signals (Friston, 2023); and Integrated Information Theory (IIT; Tononi et al., 2016), assuming that consciousness can be quantified as the integrated information which emerges in complex systems and is not reducible to the activity of its isolated parts. The recently proposed Temporo-spatial Theory of Consciousnes argues that the brain generates its own space and time based on spontaneous neuronal activity (TTC; Northoff and Zilio, 2022a). In spite of the differences between these theories they all have in common that they refer to large-scale spatiotemporal activity patterns in complex dynamic systems and are, at this stage, restricted to animals with highly differentiated central nervous systems (Mallatt et al., 2021).

While there is no global consensus on the physical correlate of consciousness, there is considerable overlap between the prevailing theories, and an integrative discussion is currently emerging (Northoff and Zilio, 2022b; Storm et al., 2024). Nevertheless, clinical measures of consciousness follow empirically constructed scales without foundation in a globally accepted theoretical concept. This is particularly unsatisfying in psychopathology which does not yet arrive at a satisfying stage of conceptual clarity and quantifiability (see section “4 System concepts of the mind–urgently needed”). This problem of conceptual precision is enhanced if references are made to mathematical approaches of systems analysis, be it information theory, graph theory, dynamic systems theory or Bayesian statistics. For example, schizophrenia has been characterized as a structurally based imbalance in information processing, in the sense of a “disconnection syndrome” (Friston and Frith, 1995) or a “misconnection syndrome” (Andreasen et al., 1999). Despite this network-theoretical framing, none of the conceptualizations provides fundamental, clinically applicable new insights toward the characterization of schizophrenia as an “association disorder,” provided more than hundred years ago by Eugen Bleuler, on the mere basis of clinical observations (comp. Peralta and Cuesta, 2011).

Thus, global concepts of brain function are not unambiguously defined and lack applicability to pressing clinical problems (see section “2 Philosophy of neuroscience–key issues”). In this situation, an epistemological perspective might be useful to assess the explanatory potential of different theories (Stefanelli, 2023; Cogitate Consortium et al., 2025). Even then, the qualia problem (Tye, 2025), i.e., the potential irreducibility of primary experiences (like pain or color perception), may remain unsolved (Nagel, 1974; Jackson, 1986). There might be a fundamental “explanatory gap” (Levine, 1983; Chalmers, 2005) between the subjective and the objective. In healthy humans, conscious (first-person) phenomena are usually expressed and accessed by others through verbal communication means. However, neuroscience has made considerable progress in developing methods to access deep layers of consciousness (e.g., the experience of pain or desire) in subjects who are unable to verbal communication including small children, severely disabled patients and possibly animals. As an example, a paradigmatic study was performed by Yu et al. (2013) who studied non-communicative humans with Unresponsive Wakefulness Syndrome (UWS) following severe brain injuries. Presenting cries of pain from other humans resulted in activation of the classical pain matrix in the brains of 24 of 44 patients, although none of them had received any painful stimuli. Whereas neurologists still discuss whether UWS patients can experience pain, such results open the possibility that many of them can feel the suffering of others. Such data indicate that “affective consciousness” can be separated from cognition, and that the former can exist in humans which have no capacity for the latter.

As long as such fundamental questions and challenges persist, we call for a critical discourse on basic concepts, arguing strategies, the very nature of neurobiological explanations and interdisciplinary interfaces within the neuroscience community. Such a conceptually based theoretical framing of neuroscience would facilitate fruitful interactions between different scientific cultures. Ultimately, it might trigger qualitative advances in the understanding of mental processes and the related disorders.

## 2 Philosophy of neuroscience–key issues

Thus, we need philosophy for assistance and a more conceptual, if not philosophical attitude within the neurosciences themself. This discourse must deal with the fragmented nature of modern neurosciences with their different research traditions, system levels, basic and applied directions. Of note, the meta-scientific reflection within philosophy is likewise fragmented into many different approaches and frameworks. Therefore, philosophy itself should strive for an integrative perspective in the interdisciplinary dialogue.

A reflection from (and interaction between) different philosophical disciplines might help clarifying epistemological weaknesses, conceptual flaws and irresolvable conundrums. Social philosophy (and social sciences) may question biases arising from contextual factors of modern neuroscience (see section “1 Some key characteristics of modern neuroscience,” and comp. Kasavir, 2023), philosophy of biology may refer to evolutionary roots of centralized nervous systems, philosophy of mind may define peculiar properties of cognitive, emotional and social processes including consciousness, and epistemology of science considers scientific knowledge production.

It should be reminded here that in ancient times, philosophy, and what is nowadays called sciences, formed unity that was most impressively personified by Aristotle. Probably the last eminent and influential connection between science and philosophy in modern times was realized about 100 years ago by the Vienna Circle with Moritz Schlick, Rudolf Carnap and others (Sigmund, 2017; Sarlkar, 2024). Together with other philosophical traditions this movement resulted in analytical philosophy with its emphasis of language as a decisive tool of thought and subject of philosophical analysis. As a result, language has become a core issue in the analysis of theory construction (see, e.g., Carnap, 1956) and has even been suggested to reflect a universal architecture underlying cognition (Chomsky, 1957; Fill and Mühlhäusler, 2001). Current knowledge in cognitive and behavioral neurobiology, however, shows that mental operations (Tolman, 1948), affective states (Panksepp, 1998, 2006) and elementary forms of consciousness (Yu et al., 2013) are possible in the absence of language. Nevertheless, pioneers of systems biology have referred to the philosophy of language in their approaches to construct theories of life (Bertalanffy, 1968; Miller, 1978).

Another important outcome of the Vienna school was logical empiricism as a foundational, anti-metaphysical principle of scientific theories. Karl Popper’s general concept of hypothesis-driven research was generated within this context. But modern science did not reach this conceptual, logical and epistemic unity. About 50 years ago the philosopher Paul Feyerabend, with his critical narrative “Against Method,” claimed for epistemological plurality. In addition, the science historian Thomas Kuhn, with his construct of “paradigms” elaborated that social mechanisms determine scientific progress (Kuhn, 1962; see also Daston, 2015). In the following, this approach of sociologically oriented “science studies” has been further developed by authors like Steve Woolgar, Bruno Latour, Helga Nowotny, Karin Knorr-Cetina and others, indicating that not so much the research logic in the tradition of Popper, but also the social practice of research shape what we regard as valid evidence (Felt and Irvine, 2014). This also seems to be true – but not sufficiently appreciated and discussed – within the neurosciences (Brown, 2019). In consequence, more recent concepts of philosophy of science are less axiomatic, but grounded in its pragmatic turn, addressing models as heuristic tools (Morgan and Morrison, 1999; Falkenburg and Hartmann, 2021) and highlighting mechanistic explanation strategies in biology and neuroscience (Machamer, 2004; Craver, 2007; Bechtel, 2008). Informed and critical accounts of the explanatory power and the limits of neuroscientific approaches would also help calibrating the impact of neurosciences on other fields of science and humanities. This might facilitate constructive inputs from the neurosciences into other areas of science and humanities like law, economics, didactics, ethics, or even theology, as reflected in the increasing use of these disciplines with the prefix “neuro-” (de Vos, 2016; Schleim, 2014).

In summary, reflections on science were initiated by philosophy but have been dominated by historians and sociologists more recently. Notwithstanding, we claim that the empirical analysis of sociocultural and socioeconomic conditions of science cannot replace philosophical reflection - we need both! In line with this necessity, a new research field in Epistemology of Science and Technology is emerging that integrates philosophical and social science perspectives (Moreno and Vinck, 2021). This has not yet, however, fully unfolded in the field of neuroscience. In particular, the recent philosophical work has not yet been consequently applied to studying the scope of models and mechanistic explanations in cognitive neuroscience.

Finally, considering this diversity and acknowledging that knowledge is “situated” (Haraway, 1988), we propose that a convergence of philosophy of mind, philosophy of (neuro)science (Bennett and Hacker, 2021), philosophy of interdisciplinarity (Schmidt, 2022) and philosophy of complex systems (Hooker, 2011) could contribute to the urgently needed integrative “Neurophilosophy” (Bickle et al., 2019; Bechtel and Huang, 2022). At present, mereological and categorical fallacies bias neuroscientific research in experiments and theories – e.g., when thoughts, emotions, beliefs or surprises are ascribed to brains, rather than to situated persons (Bennett and Hacker, 2021), or when the mechanistic explanations of neuroscience are extended from brain components to consciousness (Falkenburg, 2024). Such statements, although harmless at first sight, can give rise to an unwanted “ontological dualism/pluralism” (Pernu, 2022), whereby ontological issues regarding systems dynamics probably could be framed by “process ontology” (Nicholson and Dupré, 2018). Finally, it has to be considered that important additional contributions could come from personalized psychiatry and philosophical anthropology (Hacker, 2007, 2013, 2017, 2021) as well as from ethics (Neuroethics/Neurolaw; Schleim, 2025; see sections “5 Toward an ecological anthropology - the situated person” and “6 An important example case: the debate on “free will””).

## 3 Systems thinking–options for conceptual integration

Neuroscientists describe their results and theories frequently by terms based in dynamic system theory, like “activation,” “inhibition,” “networks,” “feedback,” “structure,” “function,” “dynamics,” “information,” “entropy,” “(non-)equilibrium,” or “oscillations.” One of the most useful systemic constructs is “(non-)equilibrium” of complex dynamic systems, often illustrated by the behavior of coupled pendula, but well applicable to complex systems like the brain’s neurochemistry (comp. Tretter, 2022). Other systemic concepts are increasingly used in the neurosciences, e.g., “self-organization,” which refers to system-internal mechanisms of emergence, and lately “resilience” addressing the full restitution of a system following perturbations.

In this epistemic context, “systems” are understood as a set of “elements” and a set of “relations” within a language of systems science (Combs, 1995). This language is used in a wide range of disciplines and may be classified as a kind of supra- or metadisciplinary terminology (Bertalanffy, 1968). Based on such terms, concepts and constructs, explicit conceptual theories and models of multi-systemic mind-brain phenomena can be developed and might facilitate a heuristically fruitful interdisciplinary dialogue between neurobiology and the sciences of the mind.

In such a context of systems thinking, the brain can be seen as an example of a complex electrochemical system with self-organized emergent and adaptive dynamics. The systemic approach, with its strong roots in physics (e.g., thermodynamics) and biology (e.g., population dynamics), allows identifying causal relations explaining the behavior of a system in a given state and with given boundary conditions. It can provide input-output analyses and (often incomplete) causal models explaining the data. It can derive relevant spatio-temporal structures, such as attractor dynamics from its models which are applicable from precise data-driven network models in molecular-cellular biology or simple neuronal networks to more abstract, simplified models of very complex physical systems like the mammalian neocortex. Such approaches comprise data-driven bottom-up models as well as conceptual top-down models (Braun et al., 2007). The required cross-disciplinarily used mathematical tools like graph theory (Barabási et al., 2023), coupled differential equations, Markov chains or matrix algebra constitute a common formal methodology of systems science (IIASA, 2025). However, these formalisms must be accompanied by qualitative, heuristic models which allow understanding, guide research strategies and improve communication between scientists. In any case, an elaborated methodology of modeling and simulation as well as prototypical models and theories are essentials of systems science (Mobus, 2023; Meadows, 2008, 2025). In this context, the formulation of models by diagrams is a significant method for communication amongst different disciplines (Sheredos et al., 2013). While some proponents of philosophy of mechanistic explanation classify concepts of systems science as “filler terms” (Craver, 2007), we believe that they offer a universal interdisciplinary language of conceptual building blocks for modeling dynamic systems at all levels of living systems (Lyre, 2018). Not at last, this level-bridging or level-integrating property is a strength of thinking in terms of dynamic systems.

## 4 System concepts of the mind–urgently needed

As described before, the brain can be characterized as a dynamic, hierarchical, and adaptive “system,” consisting of myriads of “elements” (neurons) and their “relations” (fibers). Similar systemic models can be applied to lower (e.g., cellular, biochemical) or higher (e.g., whole-brain connectome of ecological relations). However, in the sciences of the mind (e.g., Psychology, Psychotherapy, Psychiatry, Cognitive science, including Psychopathology), only few theoretical approaches try to understand mental processes and states as a multi-component multi-level processing system, an approach which could facilitate understanding brain-mind relations. Mental processes can be ordered into a macro-scale perspective of the situated mind in its natural and social environment, the meso-scale perspective of major mental capacities like perception, cognition, memory, emotions and motives and in multiple micro-level phenomena like different emotions, subtypes of memory etc. Other hierarchical organizations refer to overarching tasks (computations), cognitive strategies (algorithms) and concrete neuronal processes (biological implementations; Marr, 1982), or to biological orders from the molecular via cellular and network level to cognition and behavior (Ellis, 2018). Importantly, modern concepts of this kind include causal relations within and between all levels (including bottom-up as well as top-down causation) and parallel processing of all interacting elements at all levels (Woodward, 2003; Mitchell, 2009). These elements include basic biological processes as well as brain-body interactions or the whole organism within its natural and social environment (Fuchs, 2018, 2021).

This reserve against might be caused by the fact that academic psychology, after the strict behavioristic black box period until the 1970s, underwent a cognitive turn. This cognition-centered view (perception, thinking, memory) lasts until today. As one consequence, it implicates a relative neglect regarding other categories of the elements of the mind such as “emotions” and “motives.” In fact, all functional components of the mind should be clearly defined, and their contributions and interactions should be considered in physiological and pathological conditions. Currently, systemic models of the mind focus on cognition, albeit with some exceptions like the “affect logics” of the psychiatrist Ciompi (1988; 2025), emotional endophenotypes (Panksepp, 1998, 2006) and, more lately, clinical frameworks informed by psychoanalytic concepts such as Mark Solms’ Neuropsychoanalytic Clinical Approach (NPCA; Solms, 2021) or the Relational Affective Model (RAM; Mizen S., 2014; Mizen C. S., 2014; Mizen and Hook, 2020) that highlight the role of emotions and extend the perspective.

A new and promising systemic perspective has emerged with the comprehensive “network psychopathology” of Denny Borsboom’s group in Amsterdam (Lange et al., 2020; Lunansky et al., 2021). Other examples are Volition psychology that focusses on human action as nested control loops, the Rubicon model (Heckhausen, 2007) or lately the Personality-System-Interaction model (Kuhl and Baumann, 2021), all based on approaches from systems dynamics. Interestingly, within neurobiology, the neuronal networks related to “cognition,” “emotion,” “volition” etc., are mostly studied in isolation, i.e., without including other systemically connected mental operations. It should not be forgotten that functional elements of the mind such as perception, expectation, emotion, thinking, memory, needs, plans and (motor) behavior are intricately interconnected and should be explicitly considered as functionally connected in experimental research (Borsboom, 2017) and in theoretical models (Tretter and Löffler-Stastka, 2018).

For instance, it has been known for decades that a mismatch of perceptions related to respective expectations can lead not only to surprise and perplexity but also to negative emotions (e.g., aggression, anxiety, depression), depending on their actual difference (Dollard and Miller, 1950). It is astonishing in this context that the widely accepted Predictive Processing Theory (PPT, see section “1.3 Theoretical neuroscience - from areas to predictive networks, but what then?”), does not refer to such psychological models. It also does not consider the mindful brain as a goal-directed system. Remarkably, nevertheless PPT is widely accepted in the psychoanalytic community (Holmes, 2022).

In consequence, a systemic conception of the mind must be multi-level, must encompass multiple-loops, and should distinguish phases of predominance of mental operations and states: for instance, voluntary action (comp. section “6 An important example case: the debate on “free will””), conceived as a basic feature of the mental processing system, is constituted by states like desires (or wishes), reasoning, imagination, decision, action-planning and effect-expecting processes. In addition, in a systemic core model, the psychoanalytic concept of a “Self” and an “Ego” (and Id and Superego) could be heuristically fruitful, at least in context of a reflecting practice in treatment and prevention. In this context, the emergence of “Neuropsychoanalysis” (Kaplan-Solms and Solms, 2000; Northoff, 2023) may fruitfully integrate conceptual elements of other theories of mind, including personality functioning and narcissistic defense mechanisms (Britton, 2020; Mizen S., 2014).

In summary, such conceptual integrations within the sciences of the mind may strongly benefit from the application of dynamic system theory (Scheffer et al., 2024). A complementary and well compatible approach is to integrate the embodiment of the mind and the environmental embeddedness of persons as the brain is part of the body, and persons are organism within their ecosystem and situated subjects at the same time. These considerations are fundamental for a human-centered ecosystemic praxis in medicine (Tretter, 2008; Fuchs, 2018).

## 5 Toward an ecological anthropology - the situated person

Considering the person as a situated organism and subject contrasts with the dominant research program in the neuroscience of mental processes. Here, the focus seems to be on central molecular (including genetic) or network-level constituents of the (dys)function under study. This approach neglects the necessity for “zooming-out” and re-considering the whole organism and its environment. This loss of a holistic, physiological view should be counteracted by integrating across biological system levels and beyond (humans within their natural and social environment). One promising example might be the recent discoveries regarding nutrition, the microbiome and the gut-brain axis (Zhang et al., 2025). Another example has emerged in the context of infectious diseases, taking into account interactions of the nervous system, the endocrine system and the immune system (Tretter et al., 2021). In basic sciences, there is increasing interest in brain-body interactions through basic physiological action cycles (Draguhn and Sauer, 2023; Tort et al., 2025). Such approaches might lead to an integrated understanding of the multi-level dynamics of the organism as a dynamic yet stabilized (resilient) “system of systems.” This would result in a holistic, but differentiated and scientifically grounded understanding of the person, beyond ecologically invalid reductionism.

The above-described endeavor calls for the traditional academic discipline of anthropology in a broad, interdisciplinary and philosophically grounded sense. However, as mentioned in section “2 Philosophy of neuroscience–key issues,” it is challenging to integrate the diverse biological, psychological and socio-cultural approaches into one integrative framework. At the institutional level, there are only few organizations and universities striving for such an integrative view [e.g., BANotes.org (n.d.)]. In any case, a holistic view must consider the embeddedness of the person into the environment, i.e., the human ecological perspective. Such an eco-systemic view was influentially suggested by George Engel with his bio-psycho-social model (Engel, 1977). This concept includes cultural and societal embeddings, with a particular role for language in the formation of traditions, believes, behavioral traits, rules, and ethical bounds. An early holistic view on human communication has been proposed by Gregory Bateson’s ecology of mind which integrates approaches from anthropology, psychiatry, evolution, and epistemology (Bateson, 1972; see also Haugen, 1972). In present days, the particular role of language for cultural evolution and societal processes has to be reflected in the light of modern media which prompt the urgent need for a “media ecology” (McLuhan et al., 1967; Postman, 2009).

In times of challenges like the climate change it must now be extended to an “eco-psycho-social” or “ecosystemic” model (Stineman and Streim, 2010; Tretter and Löffler-Stastka, 2019): bodily functions like breathing and nutrition, but also interactions with the surrounding ecosystem are fully integrated into homeostatic loops of sensorimotor and social interactions (Bronfenbrenner, 1979). The related concept of persons as “situated subjects” implies a similar ecological perspective. Circular processes are also central to the theory of organisms developed by Uexküll (1973) (“function circle”). All these perspectives correspond strongly to the lately developed “circularity of the embodied mind” (Fuchs, 2020), which suggests that affective-cognitive functions arise from circular feedback loops between the brain, the entire organism, and its environment. These separated but coupled entities exhibit the property of “resonance” (Fuchs, 2018), which captures the bidirectional relationship between body and brain, as well as between organism and environment. In this approach, the brain remains central, but not solitary: it is the organ of relation, integration, and coordination within a wider sensorimotor and socio-cultural ecology.

In line with this perspective, one of the most elaborated and integrative ecological theories is labeled as the paradigm of “4E cognition.” It proposes that the mind/cognition is “embodied,” “enacted,” “extended,” and “embedded” (Varela et al., 1991; Clark and Chalmers, 1998; Gallagher, 2005, 2018; Di Paolo, 2005, 2009; see also Newen et al., 2018). This perspective rejects classical reductionist theories as well as cognition-based computer metaphorics or representational models. It also provides an appropriate contextualization of neuroscientific approaches (de Vos, 2016; Schleim, 2014), where the scientific study of brain-related mechanisms is just one (important) perspective on the embedded individual. Specifically, the 4E paradigm conceptualizes the mind and cognitive states as a dynamic system distributed across the brain, body, together with the physical and social environment (Port and van Gelder, 1995; Kelso, 1995). There are four essential conceptual building blocks:

1. “Embodiment,” which means that the body is an integral part of cognition by convergence of proprioception, interoception, and autonomic signals. In consequence, this brain-body integration leads to a “functional fusion” (Damasio, 2010).
2. “Enactedness,” which conceives cognition as an active “sense-making” by the organism, and perception as a skill, not a passive reception of external input (Noë, 2004).
3. “Extendedness” refers to cognitive processes that extend to tools, technologies, and social systems. In this view navigation, problem solving, and memory are embedded in “distributed cognitive systems” (Hutchins, 1995). The integration of external structures into cognitive loops is enabled by the plastic nature of the brain (Anderson, 2010; Menary, 2010).
4. “Embeddedness” means that cognition is embedded in physical, social, and cultural structures that shape and constrain mental processes. There is a correspondence with Gibson’s ecological psychology (Gibson, 1979) and the concept of affordances–action opportunities provided by the environment. Kirsh (1995; 2010) showed how environmental arrangement reduces internal cognitive load, for example in problem solving or skillful action. Accordingly, the brain functions as a coordinator of environmental engagement, optimized for tracking affordances, stabilizing perception through action, and integrating contextual cues.

As we can see, the 4E approach decentralizes the brain, but does not deny its importance. Rather, the 4E reinterpret its role along several lines. The brain is integration within a sensorimotor ecology including inseparable interoceptive and proprioceptive loops, movement dynamics, and environmental affordances (Gallagher, 2005). Brain plasticity enables the incorporation of natural, technological and cultural needs, skills and experiences into ever-adapting scaffolds (Menary, 2010; Clark, 2008). Social cognition often spans multiple individuals, and neural activity prepares agents for coupling with others in joint attention, dialogue, and shared tasks (De Jaegher and Di Paolo, 2007). Thus, by the 4E conception, the brain does not serve to mirror or model the external world; rather, it is the ecology of the brain (Fuchs, 2020, its active engagement with bodily, physical, and social environments, that allows for an adequate understanding of the human mind.

While we regard the approaches described in this section as very helpful and promising, much conceptual work and interdisciplinary dialogue is needed before a fully integrative view at the brain has become mainstream.

## 6 An important example case: the debate on “free will”

What has been said before can and should be related to clinical psychiatry. However, a much more frequent application are the thousands of micro-decisions to be made in everyday life. The issue of decision making is closely related to the long-standing philosophical debate about the “free will” (Kane, 2012; O’Connor, 2002; O’Connor and Franklin, 2022; Kotchoubey, 2012; Schleim, 2024; Tebartz-van Elst, 2015). This discussion, not least triggered by the famous experiments by Libet et al. (1983), has resulted in a controversial debate on personal freedom and responsibility which is still ongoing. A radical position, defended by several neuroscientists, psychologists and philosophers at the time was that any substantial concept of freedom is misled: our conscious self can merely confirm what physiological processes in the brain already determined to be done. In order to evaluate these radical interpretations of Libet’s findings (and similar later results), the concrete experimental setting of Libet should be recalled: subjects were instructed to make a voluntary finger movement whenever they felt the urge to do so, and they had to watch a clock to determine the time of their subjective decision to perform the movement. In parallel, EEGs were recorded allowing to identify the readiness potential (RP) from averaged traces. The surprising finding was that the RP preceded the so-called voluntary decision to act by several hundred milliseconds. Methodological criticisms and replication studies revealed several flaws and inconsistencies (Schmidt et al., 2016; Schurger et al., 2021; Trevena and Miller, 2002), but confirmed the general notion that neuronal processes are preceding self-experienced decisions (Soon et al., 2013).

However, an appropriate interpretation of these results should be done within volition psychology as an adequate conceptual framing, for instance by the well-established process- and phase-based “Rubicon model” (Heckhausen and Heckhausen, 2010). This empirically grounded systemic model connects motivation, volition, decision, and action by several nested feedback loops. It suggests that the volition process starts with the (predecisional) motivation and transits to (preactional) volition formation (Rubicon), which is followed by actional volition, and ends with postactional motivation. Within this framework, the timeline of observed events suggests that the RP corresponds to the transition from preactional volition (intention-formation) to actional volition (intention-initiation). The experimental setup implicates that the willingness to participate in the experiment (intention formation) already represents the Rubicon. Subsequently, the subjects get adjusted to the experimental setup and the motoric action must be learned in order to reduce noise. Therefore, during the following recording phase the RP corresponds to the model’s phase of actional volition (intention initiation).

This framing of the experimental situation implies that neuroscientific experiments like those by Libet and colleagues or Soon and colleagues only investigate a small part of the decision-making process as described by established psychological models. With regard to 4E cognition (addressed in the previous section) it should also be noted that the apparently free decisions to spontaneously move one’s finger or randomly press a button neglect the embedded and enactive nature of most or our real-world choices: that they have meaning for the agent and others, that they serve certain goals, and that they are often taken in interaction with others. At best, the described neuroscientific experiments can show that such spontaneous decisions are initiated unconsciously but based on former experiences. Libet and colleagues already pointed out that the execution of the actions can still be vetoed after the occurrence of the RP, which has been replicated in later studies.

In a yet broader context, the experiments were discussed within global frameworks of determinism or indeterminism. A strong motive in these debates was that, as the deterministic view is so successful in many areas of science, human behavior should also be conceptualized as being determined. This position overestimates the generalizability of classical physics (Cartwright, 1983), and its applicability to decision-making in humans must be debated. Also, determinism and causality should be distinguished and their use well defined within the actual context. Causation has different forms and meanings within physics (consider, e.g., the distinction between classical and quantum physics), and the concept is equally complex within the neurosciences and psychology. Indeed, most causal inferences are insufficient (Falkenburg, 2024; Hoefer, 2024), in stark contrast to expectations from simple mechanical systems (e.g., impulse transfer between two balls). Given the complexity and diversity of theories of causation, stochasticity, chaos, and emergence, interpretations of experimental results from neurophysiology and -psychology on all levels of the brain and the mind should be done with care and with explicit reference to the respective epistemological concepts and argumentative strategies (Braun et al., 1994; Braun, 2021). Especially, the essential distinction between microscopic and meso-/macroscopic systems must be considered more explicitly and precisely in this discussion (Bishop, 2002; Potter et al., 2025).

If determinism is a misleading myth that implies reductionism (Falkenburg, 2024), then the classical doctrine of free will, with its neglect of physical constraints and empirical evidence, does not offer a promising alternative either. To escape the philosophical impasse between determinism and free will, it is necessary to integrate the interdisciplinary debate on decision-making. As a beginning, such discussions should not treat “free will” as a simple yes- or no-question. And free will should not be treated as a metaphysical power, but reconceived as an emergent, trainable capacity of self-formation that encompasses neural, psychological, and phenomenological levels. Neuroscience itself may suggest that decision-making is a dynamic, probabilistic process rather than a linear chain of deterministic causes, leaving room for self-modulation and learning. Psychology and cognitive sciences demonstrate the plasticity of will through habits, attention, and self-regulation, while phenomenology reminds us that freedom is experienced as embodied self-formation within temporal and social horizons. Thus, “freedom” becomes a cultivated ability to align oneself with reasons, emotions, and actions–an art of autonomy rather than a metaphysical construct. Some philosophers (Bieri, 2001; Fuchs, 2021) show how this integrative, interdisciplinary perspective transforms the old stalemate into a progressive research program on how we actually become free. Importantly, such integrative approaches do justice to the importance of our ecological embeddedness, both physically and socially. Together, these considerations show that the debate around free will is not fully covered by neurobiological statements but requires an interdisciplinary discourse involving philosophy of sciences, philosophy of mind, and different approaches from mental and social sciences. Only within this framing can the – highly relevant! – neurobiological findings be adequately framed and understood (Brass et al., 2019; Schleim, 2024).

## 7 Conclusion

We posit that institutional mechanisms and technology-driven empirical practices within brain research might hinder the development of a sound, integrative theoretical neuroscience. An appropriate theoretical foundation must go beyond data analytics and mathematical formulations but should rather be based on a solid conceptual framework (section “1 Some key characteristics of modern neuroscience”). At present, several epistemic obstacles call for more philosophical and psychological reflection (sections “2 Philosophy of neuroscience–key issues,” “4 System concepts of the mind–urgently needed”). Parts of the conceptual gap can be filled by system theories enabling “integrated interdisciplinarity” instead of “associative interdisciplinarity” (section “3 Systems thinking–options for conceptual integration”). System theoretical elaborations of the mind by psychology and psychiatry seem also to be key to tackle the mind-brain problem in its different variants (section “4 System concepts of the mind–urgently needed”). To make neuroscientific research fruitful for problems and applications of real life, the consideration of ecology, namely the embodied and embedded mindful brain and body, might be useful (section “5 Toward an ecological anthropology - the situated person”). As a concrete application of such thinking, we highlight the importance of an integrative interdisciplinary discourse for advances in the question of free will (section “6 An important example case: the debate on “free will””).

Recent development in machine learning, robotics and artificial intelligence challenge traditional understandings of the human being and its mind, in particular the uniqueness of our mental faculties. Driven by this dynamic process reflecting concepts of the mind and its relations to other organisms and non-living natural and technical entities has become more important than ever. This urgent demand calls for the institutionalization of a broad and integrative “philosophy of neuroscience” - also including neuroethics, systems thinking, and psychology. Such efforts may foster the demanded conceptual and theoretical integration of neurosciences and support the development of new applications.

## Data Availability

The original contributions presented in this study are included in the article/supplementary material, further inquiries can be directed to the corresponding author.
